# 

**Published:** 1906-11

**Authors:** 


### BOOKS RECEIVED.

A Textbook of Histology. By Frederick R. Bailey, M.D., Adjunct Professor of Normal Histology, Medical Department of Columbia University, New York. Second, revised edition. Illustrated. Octavo, pp. 497. New York: William Wood & Co. 1906. (Price, $3.00).

The Medical Student's Manual of Chemistry. By R. A. Witthaus, M.D., Professor of Chemistry, Physics and Toxicology in Cornell University. Sixth edition. Octavo, pp. 820. New York: William Wood & Co. 1906. (Price, $4.00).

Transactions of the twenty-eighth annual meeting of the American Laryngological Association held at Niagara Falls, N. Y., May 31, June 1 and 2, 1906. James E. Newcomb, M.D., secretary.

Lea's Series of Medical Epitomes. Edited by Victor C. Pedersen, M.D. Materia Medica and Therapeutics by Edward J. Kiepe, M.D., Adjunct Professor of Materia Medica in the Medical Department, University of Buffalo. 12mo., pp. 265. Philadelphia and New York: Lea Brothers & Company. 1906. (Price, $1.00).

Annual report of the Department of Health of the City of Buffalo for the year ending December 31, 1905. Walter D. Greene, M.D., Health Commissioner.

Rhythmotherapy, or a Discussion of the Physiologic Basis and Therapeutic Potency of Mechano-vital Vibration; to which is added a Dictionary of Diseases. Illustrated by Samuel S. Wallian, A.M., M.D., Chicago: Ouellette Press. 1906. (Price, $1.50).

International Clinics. A quarterly of Illustrated Clinical Lectures and especially prepared articles on Treatment, Medicine, Surgery, Neurology, Pediatrics, Obstetrics, Gynecology, Orthopedics, Pathology, Dermatology. Ophthalmology, Otology, Rhinology, Laryngology, Hygiene and other topics of interest to students and practitioners. By leading members of the medical profession throughout the world. Edited by A. O. J. Kelly, A.M., M.D. Philadelphia. Volume III. Sixteenth series. 1906. Philadelphia and London: J. B. Lippincott Co. (Cloth, $2.00).

